# Salt for the Throat

**Published:** 1877-10

**Authors:** 


### SALT FOR THE THROAT.

An exchange says: "In these days when diseases of the throat prevail, and particularly a dry, hacking cough, which is not only distressing to ourselves, but to those with whom we are brought into business contract, those thus afflicted may be benefited by trying the following remedy : Last fall we were induced to try what virture there was in common salt. We put a teaspoonfull in about half a tumbler of cold water, and with this we gargled the throat most effectually just before meal-time. The result has been that during the winter we were not only free from the usual coughs and colds to which, as far as our memory extends, we have always been subject, but the dry, hacking cough has entirely disappeared. We attribute it entirely to the salt gargle, and do most cordially recommend it to those of our readers who are subject to diseases of the throat."

